# Genetic diversity and structure of *Oncomelania hupensis* snails in an area where *Schistosoma japonicum* transmission has been interrupted for nearly 30 years

**DOI:** 10.1051/parasite/2025031

**Published:** 2025-06-24

**Authors:** Ze-Ting Liu, Han-Qi Peng, Yu-Xin Qi, Xiao-Yan Wu, Qing Xu, Han-Xiang Zhang, Da-Bing Lu

**Affiliations:** 1 Department of Epidemiology and Statistics, School of Public Health, Jiangsu Key Laboratory of Preventive and Translational Medicine for Geriatric Diseases, MOE Key Laboratory of Geriatric Diseases and Immunology, Suzhou Medical College of Soochow University Suzhou Jiangsu 215123 P.R. China; 2 Community Health Service Center of Nicheng Pudong New Area District Shanghai 201306 P.R. China

**Keywords:** *Oncomelania hupensis*, Population genetic analyses, Microsatellites, Snail monitoring

## Abstract

China was once a major endemic zone for *Schistosoma japonicum*, but decades of control efforts have dramatically reduced transmission. Suzhou City, in Jiangsu Province, a former hyperendemic area, achieved transmission interruption in 1995. However, the intermediate host *Oncomelania hupensis* persists and new habitats in non-endemic villages pose resurgence risks if parasites are reintroduced. To evaluate genetic resilience and dispersal potential, we analyzed six *O. hupensis* populations (214 snails) from ecologically distinct habitats in Wuzhong district, Suzhou (2018 to 2021): Guangfu (GF20 and GF21: wetlands), Jinting (JT18, JT19, and JT20: isolated island), and Dongshan (DS19: lakeside hills). Using nine microsatellite loci, we identified 91 alleles and assessed genetic diversity, structure, and demography. All populations exhibited low observed heterozygosity (*Ho* < 0.5), with bottlenecks detected in GF21, GF20, and JT20. Paradoxically, infinite effective population sizes (*Ne*) at 95% CI upper limits suggested retained adaptive potential. Significant genetic differentiation (*F*_ST_ = 0.287, *p* < 0.01) reflected habitat-driven isolation: Jinting’s island populations diverged markedly from Dongshan and Guangfu, while bidirectional gene flow (*Nm* > 1) between Guangfu’s temporally sampled populations indicated sustained genetic connectivity over time. DIYABC modeling traced JT20’s ancestry to admixture between Jinting (JT18) and Guangfu (GF20) sources, implicating flood-mediated dispersal. Despite local control efficacy, snails retain resilience *via* large *Ne*. These findings mandate habitat-tailored strategies: habitat modification and intensified molluscicide campaigns in Guangfu and targeted eradication of Jinting’s isolated populations. Integrating genetic surveillance into snail monitoring programs will be critical to sustaining transmission interruption and achieving elimination in ecologically complex regions.

## Abstract (fr)

La Chine était autrefois une zone d’endémie majeure pour *Schistosoma japonicum*, mais des décennies de lutte ont permis de réduire considérablement la transmission. La ville de Suzhou, dans la province du Jiangsu, ancienne zone hyperendémique, a réussi à interrompre la transmission en 1995. Cependant, l’hôte intermédiaire *Oncomelania hupensis* persiste et la création de nouveaux habitats dans des villages non endémiques présente un risque de résurgence en cas de réintroduction du parasite. Afin d’évaluer la résilience génétique et le potentiel de dispersion, nous avons analysé six populations d’*O. hupensis* (214 mollusques) provenant d’habitats écologiquement distincts dans le district de Wuzhong, à Suzhou (2018 à 2021) : Guangfu (GF20 et GF21 : zones humides), Jinting (JT18, JT19 et JT20 : île isolée) et Dongshan (DS19 : collines au bord du lac). À l’aide de neuf loci microsatellites, nous avons identifié 91 allèles et évalué la diversité génétique, la structure et la démographie. Toutes les populations présentaient une faible hétérozygotie observée (*Ho* < 0,5), avec des goulots d’étranglement détectés dans GF21, GF20 et JT20. Paradoxalement, des tailles de population effectives infinies (*Ne*) aux limites supérieures de l’IC à 95 % suggéraient un potentiel adaptatif conservé. Une différenciation génétique significative (*F*_ST_ = 0,287, *P* < 0,01) reflétait un isolement lié à l’habitat : les populations insulaires de Jinting divergeaient nettement de Dongshan et de Guangfu, tandis qu’un flux génétique bidirectionnel (*Nm* > 1) entre les populations échantillonnées temporellement de Guangfu indiquait une connectivité génétique soutenue au fil du temps. La modélisation DIYABC a retracé l’ascendance de JT20 jusqu’au mélange entre les sources de Jinting (JT18) et de Guangfu (GF20), impliquant une dispersion médiée par les inondations. Malgré l’efficacité du contrôle local, les mollusques conservent leur résilience grâce à un *Ne* important. Ces résultats nécessitent des stratégies adaptées à l’habitat : modification de l’habitat, intensification des campagnes de lutte antimolluscicide à Guangfu et éradication ciblée des populations isolées de Jinting. L’intégration de la surveillance génétique aux programmes de surveillance des mollusques sera essentielle pour maintenir l’interruption de la transmission et parvenir à l’élimination dans les régions écologiquement complexes.

## Introduction

Schistosomiasis is an infectious disease with a tremendous impact on human health and the social economy, affecting nearly 240 million people worldwide and putting more than 700 million people at risk of infection [6]. Among several schistosome species infecting humans, *Schistosoma japonicum* is considered the most virulent due to its higher egg production, as *S. japonicum* females produce significantly more eggs daily (~5,000) than other species (*e.g.*, *S. mansoni*: ~200) triggering granulomatous inflammation, fibrosis, and organ damage [4, 23]. Moreover, the zoonotic nature of *S. japonicum* greatly complicates transmission dynamics and control efforts [26]. For example, in the Philippines because water buffalo, cattle, dogs, and a range of feral animals all contribute significantly to disease transmission, there is a need for innovative strategies to control schistosomiasis in the long term [28]. China was once severely affected by *S. japonicum*, with 11.8 million infections reported in the 1950s [39]. Over 70 years of sustained control efforts have achieved significant progress, reducing reported cases to 19,723 advanced schistosomiasis (the severe, late-stage complications) and three chronic infections in China in 2022 [45]. However, the goal of nationwide elimination by 2030 remains challenged by the persistence of the parasite’s sole intermediate host, *Oncomelania hupensis* [10].

*Oncomelania hupensis*, a freshwater amphibious snail, inhabits slow-moving water bodies, irrigation channels, and marshlands with dense vegetation. Its distribution is tightly linked to specific ecological conditions, including humidity, temperature, and soil composition [48]. The snail’s limited dispersal capacity restricts gene flow between isolated populations, promoting genetic differentiation; however, passive dispersal *via* water currents, flooding, or human activity (*e.g.*, agricultural or water projects) can facilitate long-distance migration, particularly in connected habitats [5]. *Oncomelania hupensis* reproduces oviparously, depositing egg masses in moist substrates, with its lifetime (1–1.5 years) and population density highly sensitive to environmental disturbances such as molluscicide application or habitat modification [48]. China’s schistosomiasis control program has historically prioritized snail control and elimination through habitat modification, molluscicide application, and environmental management to disrupt *O. hupensis* breeding and reproduction [5, 34]. These strategies aim to directly suppress snail populations by fragmenting habitats and reducing hydrological connectivity, which drives genetic bottlenecks and differentiation [34]. For example, levee construction along the Yangtze River has reduced flooding frequency, isolating snail populations and eroding diversity in formerly connected wetlands [5]. The biological traits of *O. hupensis* shape expectations for its genetic structure: isolated populations may exhibit low diversity due to drift and inbreeding, while hydrologically connected habitats could sustain gene flow and resilience [20], and the capacity of *O. hupensis* populations to recover from demographic bottlenecks or environmental disturbances, while retaining genetic diversity and adaptive potential.

In China, *S. japonicum* transmission is now largely confined to regions south of the Yangtze River. Suzhou city, Jiangsu province, a former hyperendemic area on the lower reaches of the river, achieved transmission interruption in 1995 through integrated control measures [22]. Despite this success, *O. hupensis* persists in Suzhou, with new habitats recently detected in non-endemic villages of Wuzhong district [47]. This resurgence raises concerns, as genetic studies indicate shared mtDNA-based haplotypes between Suzhou snails and populations in *S. japonicum*-endemic regions like Shitai of Anhui [44], where wild rodents serve as main reservoirs [18, 24] and infected snails have been constantly reported [43]. Reintroduction of the parasite, through infected migratory workers or tourists from domestic areas or abroad [37], could reestablish transmission, as reported in Corsica where imported schistosomes caused an unexpected outbreak of urogenital schistosomiasis with more than 120 local people or tourists infected [2, 3]. This necessitates vigilant monitoring of snail population dynamics.

To evaluate the genetic resilience and dispersal potential of *O. hupensis*, we sampled six populations from three towns in Wuzhong District, Suzhou, from 2018 to 2021. Sampling targeted ecologically distinct habitats: (1) Jinting (JT): an island in Taihu Lake, isolated by up to 10 km of open water from mainland sites, with historical snail persistence despite intensive control; (2) Guangfu (GF): a wetland region interconnected by canals and seasonal floods, enabling passive snail dispersal; and (3) Dongshan (DS): a lakeside hilly area adjacent to Jinting. Temporal sampling (2018–2021) in Jinting (JT18, JT19, JT20) and Guangfu (GF20, GF21) allowed assessment of genetic stability under sustained control pressure. Using nine microsatellite loci [33], we analyzed genetic diversity, structure, and demographic history to address two questions: does habitat connectivity (*e.g.*, GF’s wetlands *vs.* JT’s isolation) drive differences in genetic diversity and gene flow? Have prolonged control efforts eroded adaptive potential, or do populations retain resilience (as measured by genetic diversity, gene flow, and demographic stability) *via* migration or cryptic refugia? This study provides critical insights into how *O. hupensis* biology and landscape ecology interact to shape genetic patterns in a region post-schistosome transmission interruption, informing snail control strategies.

## Materials and methods

### Sampling snails

**Figure 1:**
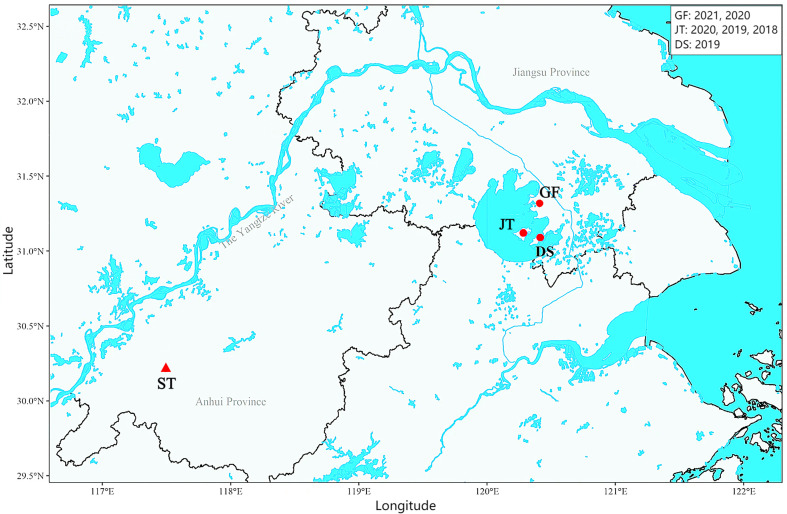
Geographical distribution of *Oncomelania hupensis* sampling in Wuzhong District, Suzhou, China. GF, JT, and DS for Guangfu, Jinting, and Dongshan, respectively. ST, for Shitai county of Anhui province, where *S. japonicum* transmission persists in wild animals.

Six populations of *O. hupensis* snails were sampled between 2018 and 2021 from three ecologically distinct towns in Wuzhong District, Suzhou: Dongshan (DS19, 2019), Guangfu (GF20, 2020; GF21, 2021), and Jinting (JT18, 2018; JT19, 2019; JT20, 2020). Given the potential for local population turnover (*e.g.*, molluscicide-driven extinctions or annual recolonization), each year’s sample was treated as an independent population to assess temporal genetic stability. All snails were confirmed to be non-infected with *S. japonicum* in the laboratory *via* cercarial shedding method [9], and only adults were preserved in ethanol for genotyping. Geographic coordinates of the targeted towns are detailed in Figure 1 and Table 1.

**Table 1:** Geographical sampling details of *Oncomelania hupensis* populations across three towns (Dongshan, Guangfu, and Jinting) in Wuzhong District, Suzhou, China (2018–2021).

Population code	Town	Latitude (N)	Longitude (E)	Year	Sample size
GF21	Guangfu	31.314645	120.410027	2021	30
GF20				2020	31
JT20	Jinting	31.120675	120.282633	2020	29
JT19				2019	43
JT18				2018	41
DS19	Dongshan	31.090821	120.413247	2019	40

### DNA extraction and microsatellite genotyping

A total of 30–45 snails were randomly selected from each snail population. Approximately 30 mg of muscle tissues from the pleopod was cut from each snail, and DNA was individually extracted using an EZgene™ Mollusc gDNA Kit (Biomiga, San Diego, CA, USA), following the manufacturer’s protocols.

PCR amplifications were conducted using a Multiplex PCR Plus Kit (QIAGEN, Hilden, Germany). Each snail was genotyped using nine previously characterized microsatellite loci (*i.e.*, T6-17, DH02, B14, C22, DH01, T4-25, D11, T1-10, and T4-22) [16, 32, 46]. The forward primer for each pair was labeled with one of four fluorescent dyes (*i.e.*, 6-FAM, HEX, TAMRA, or ROX), and two separate multiplex PCRs were employed with the first five loci in reaction one and the last four in reaction two [32]. Each PCR amplification was performed in 15 μL reactions, including 1.5 μL of snail DNA. The PCR amplification conditions were as follows: 95 °C for 5 min; 95 °C for 30 s, 60 °C for 60 s, 72 °C for 30 s, with 30 cycles; 65 °C for 30 min and 4 °C indefinitely. PCR amplification products were genotyped using an ABI3100 sequencer at Sangon Biotech (Shanghai, China).

### Data analysis

#### Genetic diversities

The accurate lengths of amplified microsatellite DNA fragments were determined using GeneMarker HID V2.6 [19] and subsequently exported to an Excel table. To assess genetic diversity, GenAlex V6.5 [29] was used to calculate the number of observed alleles (*Na*), number of effective alleles (*NeA*), observed heterozygosity (*Ho*), expected heterozygosity (*He*), and inbreeding coefficient (*F*_IS_) per locus, as well as to test deviations from Hardy-Weinberg Equilibrium (HWE) for each locus.

#### Bottleneck effect and effective population size

BOTTLENECK V1.2 [30] was used to determine whether a snail population experienced a bottleneck effect or exhibited an expansion trend. Tests were performed under two different mutational models: the Infinite Allele Model (IAM) and the Two-Phase Model (TPM), the latter of which has been shown to be more suitable for microsatellite data [38]. As only nine loci were used, Wilcoxon’s sign-rank test was applied for statistical analysis. When a bottleneck effect occurs, the number of alleles decreases faster than heterozygotes over generations. Therefore, a bottleneck effect is indicated by the presence of excess heterozygosity, whereas a deficiency in heterozygosity may indicate recent population expansion [7].

To ensure robustness, NeEstimator [11] and LDNe [41] were used to estimate the effective population size (*Ne*) and its 95% confidence intervals (CIs) for each snail population. The linkage disequilibrium (LD) method was chosen for their calculation when the alleles had a frequency greater than or equal to 0.05. *Ne* refers to the number of parental individuals who effectively contribute to the next generation, with a larger *Ne* value indicating higher genetic diversity.

#### Population genetic structure

The Analysis of Molecular Variance (AMOVA) was conducted using Arlequin V3.5 [14] to partition the sources of genetic variation within and among snail populations. *F*_ST_ was calculated and its significance was tested at the significant level of 0.01 [42]. The following quantitative guidelines were used to interpret *F*_ST_ in terms of genetic differentiation: 0 to 0.05 (as little), 0.05 to 0.15 (moderate), 0.15 to 0.25 (great), and >0.25 (very great genetic differentiation) [35].

Bayesian clustering analysis was performed using STRUCTURE V2.3 [31]. Simulations were run with a 10,000 burn-in-period and 10,000 Markov Chain Monte Carlo (MCMC) iterations. The software was run with the number of clusters (*K*) ranging from two to ten, with 20 independent runs performed for each *K* value. The result files from STRUCTURE were visually analyzed using the online STRUCTURE HARVESTER [12], from which the estimation results including ln(*K*), delta *K* and the most likely number of clusters *K* [13] were finally estimated and outputted.

Principal Coordinate Analysis (PCoA) of genetic relatedness was performed using GenAlex V6.5 [29]. The genetic distances between snail individuals were calculated and used as input for PCoA analysis. Cluster analysis was conducted using the unweighted pair group method with arithmetic means (UPGMA), and the phylogenetic tree of six snail populations was constructed using MEGA X [21].

#### Genetic relationship between populations

The gene flow (*Nm*) between six snail populations was assessed using Migrate-N V5.0.4 [1]. The following parameters were used in the calculation: the Brownian motion model, the maximum likelihood method, three long chains set to 500,000, 10 short chains set to 10,000, a burn-in value set to 10,000, and a total of 3 million iterations. The temperatures (*T*) of the hot chains used to estimate likelihood approximations were 1.0, 1.5, 3.0, and 1,000,000. To minimize error, three independent repetitions of the operation were performed. Higher *Nm* values (>1) indicate sufficient genetic exchange to counteract genetic drift and maintain population connectivity, while lower values (<1) suggest limited gene flow and potential genetic differentiation [15].

#### Population divergence history

By using DIYABC V2.1.0 [8], the historical divergence and dynamics of snail populations were first analyzed based on linear discriminant analysis of summary statistics, and then the optimal model with the highest posterior probability was estimated with logistic regression. For each assumed scenario, we employed uniform priors for all parameters (Supplementary Table S1), simulated 10^6^ datasets, calculated the posterior probability and its 95% CI, and selected the scenario with the highest posterior probability.

## Results

### Genetic diversity

Six populations comprising 214 *O. hupensis* snails were genotyped across nine microsatellite loci. The T4-22 locus was excluded due to excessive missing data from amplification failure, leaving eight loci for analysis. A total of 91 alleles were identified, with an average of 11.375 alleles per locus. Allelic diversity ranged from seven alleles at locus T6-17 to 23 alleles at locus T4-25, reflecting variability across markers.

Genetic diversity metrics across the six *O. hupensis* populations revealed marked variability (Table 2). The number of observed alleles per locus (Na) ranged from 2.625 (JT19) to 6.375 (GF21), while effective alleles (NeA) varied between 1.945 (JT19) and 3.993 (GF20). Guangfu populations exhibited the highest diversity, with GF20 showing the greatest expected heterozygosity (*He* = 0.740; unbiased *He* = 0.758) and GF21 displaying the highest observed heterozygosity (*Ho* = 0.477). In contrast, Jinting’s JT19 population had the lowest diversity (*He* = 0.356, *Ho* = 0.182). Inbreeding coefficients (F_IS_) ranged from 0.337 (GF21) to 0.596 (JT19), reflecting moderate-to-severe inbreeding. Deviations from Hardy-Weinberg Equilibrium (HWE) were widespread (64.58% of loci, *p* < 0.01), with JT19 departing from equilibrium at all loci.

**Table 2:** Genetic diversity indices for six *Oncomelania hupensis* populations.

Population		*Na*	*NeA*	*Ho*	*He*	*uHe*	*F*_IS_ (*p*-value)
GF21	Mean	6.375	3.751	0.477	0.713	0.727	0.337 (<0.001)
	SE	0.625	0.357	0.077	0.031	0.031	0.097
GF20	Mean	6.125	3.993	0.464	0.740	0.758	0.376 (<0.001)
	SE	0.718	0.288	0.066	0.020	0.022	0.085
JT20	Mean	3.375	2.338	0.313	0.560	0.571	0.479 (<0.001)
	SE	0.420	0.145	0.088	0.029	0.029	0.143
JT19	Mean	2.625	1.945	0.182	0.356	0.361	0.596 (<0.001)
	SE	0.565	0.371	0.073	0.106	0.107	0.095
JT18	Mean	3.125	2.025	0.247	0.386	0.392	0.527 (<0.001)
	SE	0.581	0.387	0.118	0.097	0.099	0.188
DS19	Mean	4.500	2.754	0.329	0.515	0.521	0.384 (<0.001)
	SE	1.052	0.456	0.114	0.114	0.116	0.139

Note: *Na*, No. of different alleles per locus; *NeA*, No. of effective alleles per locus; *Ho*, observed heterozygosity; *He*, expected heterozygosity; *uHe*, unbiased expected heterozygosity; *F*_*IS*_, the inbreeding coefficient; SE, standard error.

### Population bottleneck tests and effective population size

Bottleneck analysis (Table 3) revealed no significant heterozygosity deficiency under either the Infinite Allele Model (IAM) or Two-Phase Model (TPM) (*p* > 0.05), indicating no evidence of recent population expansions. However, significant heterozygosity excess, a signature of demographic bottlenecks, was detected in six populations under IAM (*p* < 0.05), with three populations (GF21, GF20, JT20) also showing significance under TPM (*p* < 0.05). These results strongly suggest recent bottlenecks in Guangfu (GF21, GF20) and Jinting (JT20) populations.

**Table 3:** Bottleneck test results for *Oncomelania hupensis* populations under the Infinite Allele Model (IAM) and Two-Phase Model (TPM).

Population	Probability for heterozygosity excess		Probability for heterozygosity deficiency	
	IAM	TPM	IAM	TPM
GF21	0.00195	0.00195	1.00000	1.00000
GF20	0.00195	0.00195	1.00000	1.00000
JT20	0.00195	0.00586	1.00000	0.99609
JT19	0.02344	0.05469	0.98438	0.96094
JT18	0.03906	0.46875	0.97266	0.59375
DS19	0.01563	0.21875	0.99219	0.92188

The effective population size (*Ne*) and its 95% CIs estimated using NeEst and LDNe are shown in Table 4. Infinite *Ne* for most populations (*e.g.*, GF21, JT19, JT18), reflecting retained genetic diversity and low drift. Negative *Ne* values (*e.g.*, GF21: −33.0) are methodological artifacts, interpreted as infinite due to minimal drift signals [14]. Finite estimates (*e.g.*, GF20: 64.7; DS19: 164.2) still show high upper bounds (Infinite), suggesting resilience.

**Table 4:** Effective population size (*Ne*) estimates and 95% confidence intervals for *Oncomelania hupensis* populations using NeEst and LDNe linkage disequilibrium methods.

Population	with NeEst	with LDNe
GF21	Infinite (107.9, Infinite)	−33.0 (−63.1, Infinite)
GF20	64.7 (26.9, Infinite)	−15.6 (−20.9, Infinite)
JT20	67.2 (14.0, Infinite)	276.4 (17.5, Infinite)
JT19	Infinite (59.3, Infinite)	Infinite (64.2, Infinite)
JT18	Infinite (30.8, Infinite)	Infinite (12.6, Infinite)
DS19	164.2 (34.8, Infinite)	223.2 (37.1, Infinite)

### Population structure

The nested AMOVA results (Table 5) showed significant genetic differentiation both among towns (13.22%, *F*_ST_ = 0.2874) and among years within towns (18.06%, *F*_SC_ = 0.2081). Pairwise *F*_ST_ values between snail populations (Table 6) ranged from 0.036 to 0.409 (all *p* < 0.01), with the lowest between GF20 and GF21 and the highest between DS19 and JT18.

**Table 5:** Hierarchical Analysis of Molecular Variance (AMOVA) partitioning genetic variation among towns, years within towns, and within populations.

Source of variation	df	Sum of squares	Percentage of variation	*F*-statistics
Among towns	2	82.795	13.22	*F*_ST_ = 0.2874*
Among years within towns	3	53.453	18.06	*F*_SC_ = 0.2081*
Within populations	422	388.268	68.72	
Total	427	524.516	100	

*p-value < 0.01.

**Table 6:** Pairwise *F*_ST_ values (lower triangle) and corresponding *p*-values (upper triangle) quantifying genetic differentiation among six *Oncomelania hupensis* populations.

	GF21	GF20	JT20	JT19	JT18	DS19
GF21		0.009	0.000	0.000	0.000	0.000
GF20	0.036		0.000	0.000	0.000	0.000
JT20	0.286	0.269		0.000	0.000	0.000
JT19	0.294	0.272	0.342		0.000	0.000
JT18	0.340	0.318	0.354	0.218		0.000
DS19	0.260	0.244	0.250	0.310	0.409	

**Figure 2:**
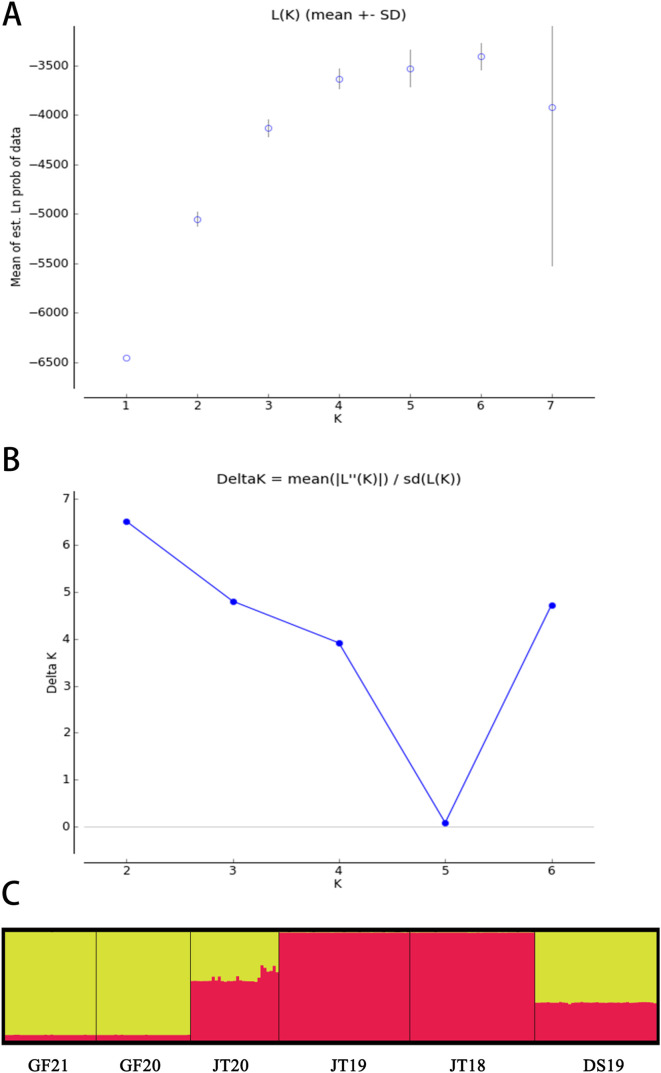
Bayesian clustering analysis (STRUCTURE) of *Oncomelania hupensis* populations, indicating optimal genetic clusters (*K* = 2). A: Mean (±SD) natural logarithm of the likelihood of the data [LnP(X | K)] for value of assumed clusters (*K*). B: Delta *K* value is plotted against the number of assumed *K*. C: Genetic structure with K = 2. Each individual is represented by a thin vertical line, which is partitioned into K colored segments that represents the individual’s estimated membership fractions in *K* clusters. Black lines separate individuals of different populations. The figure shown for a given *K* is based on the highest probability run at that *K*. Populations are labelled below the figure.

**Figure 3:**
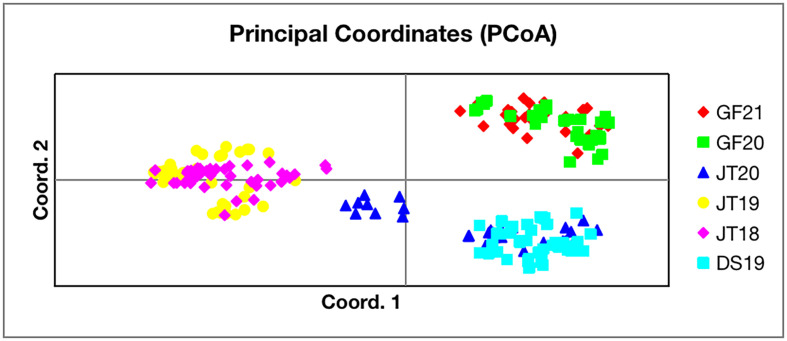
Principal Coordinate Analysis (PCoA) of genetic distances among six *Oncomelania hupensis* populations.

**Figure 4:**
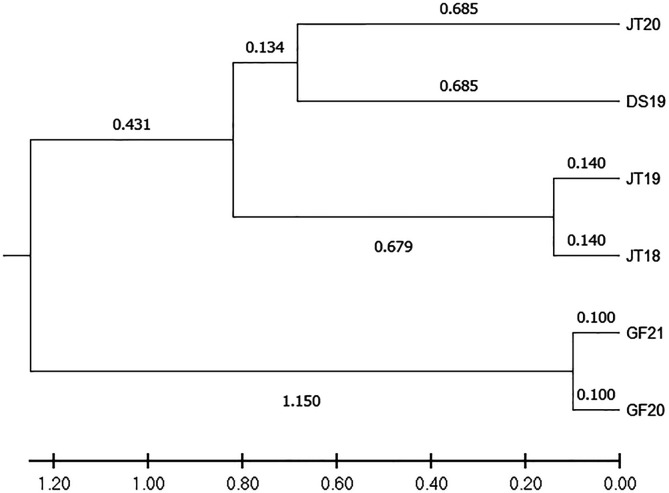
UPGMA phylogenetic tree illustrating genetic relationships among six *Oncomelania hupensis* populations.

Bayesian clustering analysis (STRUCTURE) revealed the genetic structure of the six *O. hupensis* populations (Fig. 2), with the optimal number of clusters determined as *K* = 2 based on the highest Δ*K* value (Fig. 2B). Principal Coordinate Analysis (PCoA) further supported spatial clustering, with individuals from each town forming distinct groups (Fig. 3). Notably, a subset of JT20 snails clustered with DS19, suggesting potential dispersal or admixture between Jinting’s island and Dongshan’s lakeside habitats. Phylogenetic analysis (UPGMA tree, Fig. 4) resolved populations into two primary clades: one comprising Jinting (JT18, JT19, JT20) and Dongshan (DS19), and the other containing Guangfu’s wetland populations (GF20, GF21). Within the first clade, JT20 and DS19 formed a subcluster, distinct from JT18 and JT19, reflecting finer-scale divergence.

### Populations genetic relationship and divergence history

The magnitude of gene flow calculated by Migrate-N is shown in Table 7. High gene flow (bidirectional migration, *Nm* = 1.24 to 2.32) was observed between GF21 and GF20. Minimal gene flow (*Nm* < 0.1) was observed for most other comparisons. GF21 and GF20 received the highest total migration (*Nm* = 1.25 to 2.34), while the other four populations were largely isolated (*Nm* = 0.09 to 0.27).

**Table 7:** Gene flow (*Nm*) and scaled effective population size (*θ*) estimates between *Oncomelania hupensis* populations derived from Migrate-N analysis.

Population, i	*θ*	Nm(GF21 → i)	Nm(GF20 → i)	Nm(JT20 → i)	Nm(JT19 → i)	Nm(JT18 → i)	Nm(DS19 → i)	Nm(Total → i)
GF21	1.6180		1.2389	0.0024	0.0049	0.0027	0.0049	1.2538
GF20	1.0820	2.3210		0.0073	0.0032	0.0028	0.0041	2.3385
JT20	0.3246	0.0489	0.0441		0.0046	0.0080	0.0086	0.1142
JT19	0.2172	0.0740	0.0552	0.0215		0.0418	0.0055	0.1980
JT18	0.1668	0.0465	0.0303	0.0081	0.1833		0.0059	0.2741
DS19	0.1763	0.0299	0.0490	0.0027	0.0032	0.0028		0.0876
Total		2.5205	1.4174	0.0420	0.1992	0.0581	0.0289	

Note: Population, i: represents the recipient population. *θ* (Theta): represents the scaled effective population size for population i, and quantifies genetic diversity, with higher values indicating larger populations, faster mutation rates, or both. *Nm*: represents the number of migrants per generation and quantifies gene flow between populations. *Nm* (Total → i): Total gene flow into population i from all other populations combined.

**Figure 5:**
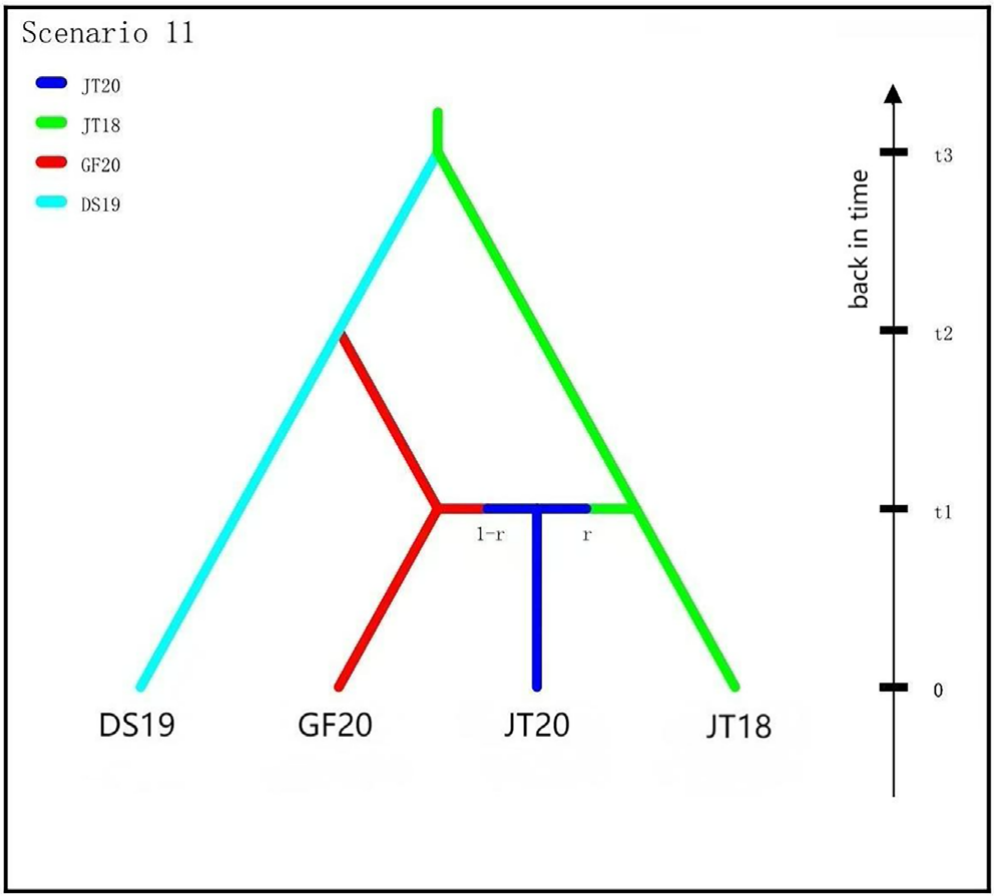
Historical divergence model (DIYABC) for four *Oncomelania hupensis* populations (GF20, DS19, JT20, and JT18), with posterior probabilities supporting admixture and split events. t3, t2, and t1 for divergence time point, and defined t3 > t2 and t2 > t1.

Given the highest gene flow observed between Guangfu populations GF21 and GF20, alongside the notably low genetic diversity in Jinting’s JT19 population, we excluded GF21 and JT19 to focus on historical divergence among four populations: JT18, DS19, JT20, and GF20. Using DIYABC V2.1.0, we modeled divergence dynamics of the four populations with JT18 (the earliest temporal population, sampled in 2018) as the ancestral population. We evaluated 33 possible historical scenarios (Supplementary Table S2), including admixture and non-admixture hypotheses, through one million simulations per scenario (Supplementary Table S3). Initial analysis identified five top-performing scenarios (3, 11, 24, 25, and 33) for further refinement. In subsequent rounds, Scenarios 11 and 24 exhibited comparable posterior probability, prompting a final evaluation. Ultimately, Scenario 11 (Supplementary Table S4) achieved the higher posterior probability (0.5594; 95% CI: 0.4551–0.6637), supporting a divergence pathway in which JT18 first split into DS19, followed by DS19 differentiating into GF20. JT20 emerged later *via* admixture between JT18 and GF20 populations (Fig. 5).

## Discussion

This study investigated the genetic diversity and population structure of six *O. hupensis* populations across three towns in Wuzhong district, Suzhou, China, sampled between 2018 and 2021. We observed markedly reduced genetic diversity in all populations, with observed heterozygosity (*Ho*) values consistently below 0.5. These values are significantly lower than those reported in other endemic regions, such as Anhui, Sichuan, and Yunnan provinces [36, 46]. The observed genetic bottlenecks, particularly in Guangfu (GF21, GF20) and Jinting (JT20), align with control efforts (*e.g.*, annual molluscicide application) implemented in all sampled sites upon snail detection, a routine practice in Suzhou since achieving transmission interruption in 1995 [17]. These interventions likely fragmented populations, reducing gene flow and accelerating genetic drift.

Genetic variation analysis revealed higher diversity within populations than among them, with 64.58% of loci deviating from Hardy-Weinberg Equilibrium (HWE), likely due to inbreeding (*F*_IS_ > 0.5 in Jinting populations) and sustained molluscicide pressure [26]. Guangfu populations (GF20, GF21) exhibited comparatively high genetic diversity (*He* ≈ 0.7) and bidirectional gene flow (*Nm* > 1), alongside infinite *Ne* estimates. These metrics collectively indicate retained adaptive potential, a capacity for rapid population recovery due to large *Ne* and sustained genetic connectivity over time, which necessitates intensified control efforts (*e.g.*, increased molluscicide frequency, habitat disruption) to counteract resilience. Conversely, Jinting populations (JT18, JT19) showed low diversity (*Ho* < 0.3) and severe inbreeding (*F*_IS_ > 0.5), likely due to geographic isolation and control-driven fragmentation. While inbreeding may elevate local extinction risks, it also increases the likelihood of drug resistance fixation in surviving snails, complicating eradication [25, 27].

Significant genetic differentiation (*F*_ST_ = 0.2874, *p* < 0.01) among populations reflects synergistic pressures from sustained control measures and landscape-mediated isolation. Jinting’s island populations (JT18, JT19, and JT20), situated ~10 km offshore in Taihu Lake, diverged markedly from lakeside Dongshan (DS19) and wetland Guangfu (GF20 and GF21). This divergence aligns with isolation-by-environment principles [40], where distinct habitats (hilly *vs.* wetland) limit dispersal and adaptive gene flow. Guangfu’s wetland facilitated high gene flow between temporally sampled populations (GF20/GF21: *Nm* > 1), while Jinting’s geographic isolation amplified drift, compounded by temporal shift resulted from annual molluscicide applications. These patterns highlight how habitat heterogeneity and control intensity interact to shape genetic structure.

Notably, JT20 individuals exhibited mixed ancestry, clustering partially with DS19, despite Jinting’s isolation. DIYABC modeling supports JT20’s origin as an admixed population derived from Jinting (JT18) and Guangfu (GF20) sources. We posit that seasonal flooding in Taihu Lake occasionally transports snails from Dongshan’s lakeside habitats to Jinting’s island, where survivors from prior control efforts (refugia) interbreed with migrants. Meanwhile, divergence modeling indicates DS19 diverged earlier from ancestral JT18, with GF20 later splitting from DS19, a chronology consistent with Jinting’s historical role as a persistent snail reservoir. These dynamics underscore the dual role of natural dispersal (*e.g.*, flooding) and human activity (*e.g.*, habitat modification) in redistributing snails, complicating elimination efforts in hydrologically dynamic regions.

While skewed sex ratios could theoretically reduce genetic diversity, the inferred infinite *Ne* and high *F*_IS_ in Jinting populations likely reflect habitat fragmentation and control-induced bottlenecks rather than reproductive skew. Future studies should integrate sex ratio validation to refine genetic monitoring and demographic history resolution for *O. hupensis* populations.

## Conclusion

This study revealed critical insights into the genetic resilience of *O. hupensis* in post-*S. japonicum* transmission interruption areas in Suzhou, China. Despite sustained control efforts, Guangfu’s wetland populations (GF20 and GF21) retained high genetic diversity and bidirectional gene flow, indicating sustained genetic connectivity over time; moreover, infinite *Ne* estimates suggest latent adaptive potential, necessitating intensified regional molluscicide campaigns. In contrast, Jinting’s isolated island populations (JT18, JT19, and JT20) exhibited low diversity and inbreeding, yet DIYABC modeling traced their origin to ancestral Jinting snails, highlighting risks of reseeding *via* flooding or human activity. We therefore recommend: (1) wetland-specific strategies: prioritize habitat modification and intensified molluscicide use in Guangfu; (2) targeted eradication: exploit Jinting’s isolation for focused elimination while monitoring flood-mediated dispersal; and (3) genetic surveillance: track *Ne* and *F*_ST_ trends to preempt resurgence. By aligning control efforts with landscape-driven genetic resilience, this work provides a roadmap for snail control and elimination in ecologically complex regions.

## Supplementary files

The supplementary material of this article is available at https://www.parasite-journal.org/10.1051/parasite/2025031/olm. *Table S1*. Parameters and prior distributions used in DIYABC analysis. *Table S2*. Demographic history scenarios assumed among four snail populations. *Table S3*. Posterior probabilities of the assumed demographic history scenarios evaluated in DIYABC analysis. *Table S4*. Posterior distributions of population demographic parameters from Scenario 11 with the highest posterior probability inferred in DIYABC analysis.
