# Supplementary material for: Genetic diversity and structure of Oncomelania hupensis snails in an area where Schistosoma japonicum transmission has been interrupted for nearly 30 years
Source: Parasite. 2025 Jun 24;32:38. doi: 10.1051/parasite/2025031 (PMC12187067; doi:10.1051/parasite/2025031)
Supplement: Supplementary file 1 — Table S1. Parameters and prior distributions used in DIYABC analysis. Table S2. Demographic history scenarios assumed among four snail populations. Table S3. Posterior probabilities of the assumed demographic history scenarios evaluated in DIYABC analysis. Table S4. Posterior distributions of population demographic parameters from Scenario 11 with the highest posterior probability inferred in DIYABC analysis. [file parasite-32-38-s1.pdf]

Table S1. Parameters and prior distributions used in DIYABC analysis

Parameter	Distribution	Interval
Effective population size, Ni	Uniform	10-10000
Rates of admixture, ra	Uniform	0.001-0.999
Mean mutation rate (MEANMU)	Uniform	10^{-4}-10^{-3}
Mean parameter of the geometric distribution of the number of repeats (MEAN P)	Uniform	0.1-0.3
Mean single nucleotide insertion/deletion mutation rate (MEANSNI)	Log-uniform	10^{-8}-10^{-5}

Table S2 Demographic history scenarios assumed among four snail populations

Analysis	Number	Scenario		
		t3	t2	t1
No admixture（t1 t2 t3)	1	JT18→DS19	JT18→GF20	JT18→JT20
	2	JT18→DS19	JT18→GF20	DS19→JT20
	**3**	**JT18→DS19**	**JT18→GF20**	**GF20→JT20**
	4	JT18→DS19	DS19→GF20	JT18→JT20
	5	JT18→DS19	DS19→GF20	DS19→JT20
	6	JT18→DS19	DS19→GF20	GF20→JT20
Admixture（t1 t2 t3)	7	JT18→DS19	JT18→GF20	JT18+DS19→JT20
	8	JT18→DS19	JT18→GF20	JT18+GF20→JT20
	9	JT18→DS19	JT18→GF20	DS19+GF20→JT20
	10	JT18→DS19	DS19→GF20	JT18+DS19→JT20
	**11**	**JT18→DS19**	**DS19→GF20**	**JT18+GF20→JT20**
	12	JT18→DS19	DS19→GF20	DS19+GF20→JT20
	13	JT18→DS19	JT18+DS19→GF20	JT18→JT20
	14	JT18→DS19	JT18+DS19→GF20	DS19→JT20
	15	JT18→DS19	JT18+DS19→GF20	GF20→JT20
	16	JT18→DS19	JT18+DS19→GF20	JT18+GF20→JT20
	17	JT18→DS19	JT18+DS19→GF20	DS19+GF20→JT20
No admixture（t1 t2)	18		JT18→DS19; JT18→GF20	JT18→JT20

	19	JT18→DS19; JT18→GF20	DS19→JT20
	20	JT18→DS19; JT18→GF20	GF20→JT20
	21	JT18→DS19	JT18→GF20; JT18→JT20
	22	JT18→DS19	DS19→GF20; JT18→JT20
	23	JT18→DS19	JT18→GF20; DS19→JT20
	**24**	**JT18→DS19**	**DS19→GF20; DS19→JT20**
Admixture（t1 t2)	**25**	**JT18→DS19; JT18→GF20**	**JT18+DS19→JT20**
	26	JT18→DS19; JT18→GF20	DS19+GF20→JT20
	27	JT18→DS19; JT18→GF20	JT18+GF20→JT20
	28	JT18→DS19	JT18→GF20; JT18+DS19→JT20
	29	JT18→DS19	DS19→GF20; JT18+DS19→JT20
	30	JT18→DS19	JT18+DS19→GF20；JT18→JT20
	31	JT18→DS19	JT18+DS19→GF20；DS19→JT20
	32	JT18→DS19	JT18+DS19→GF20；JT18+DS19→JT20
No admixture (t1)	33		JT18→DS19；JT18→GF20；JT18→JT20

Table S3 Posterior probabilities of the assumed demographic history scenarios evaluated in DIYABC analysis

Analysis	Number	Posterior Probability	95% CI
No admixture（t1 t2 t3)	1	0.0033	[0.0000,0.0785]
	2	0.0010	[0.0000,0.0778]
	**3**	**0.0231**	**[0.0000,0.0890]**
	4	0.0124	[0.0000,0.0825]
	5	0.0119	[0.0000,0.0813]
	6	0.0094	[0.0000,0.0803]
Admixture（t1 t2 t3)	7	0.0071	[0.0000,0.0796]
	8	0.0103	[0.0000,0.0808]
	9	0.0014	[0.0000,0.0779]
	10	0.0388	[0.0000,0.1025]
	**11**	**0.0759**	**[0.0009,0.1509]**
	12	0.0076	[0.0000,0.0796]
	13	0.0219	[0.0000,0.0872]
	14	0.0138	[0.0000,0.0831]
	15	0.0432	[0.0000,0.1100]
	16	0.0088	[0.0000,0.0802]
	17	0.0225	[0.0000,0.0876]
No admixture（t1 t2)	18	0.0110	[0.0000,0.0810]
	19	0.0640	[0.0000,0.1357]
	20	0.0296	[0.0000,0.0919]
	21	0.0274	[0.0000,0.0928]
	22	0.0320	[0.0000,0.1975]
	23	0.0042	[0.0000,0.0787]
	**24**	**0.1754**	**[0.0000,0.3691]**
Admixture（t1 t2)	**25**	**0.1002**	**[0.0052,0.1952]**
	26	0.0261	[0.0000,0.0886]
	27	0.0379	[0.0000,0.0988]
	28	0.0033	[0.0000,0.0784]
	29	0.0610	[0.0000,0.1307]
	30	0.0046	[0.0000,0.0790]
	31	0.0887	[0.0000,0.1925]
	32	0.0188	[0.0000,0.0861]
No admixture(t1)	33	0.0035	[0.0000,0.0784]

**The second round**			
No admixture VS Admixture	3	0.0249	[0.0000,0.1106]
	**11**	**0.3545**	**[0.2393,0.4697]**
	**24**	**0.3443**	**[0.2104,0.4783]**
	25	0.0690	[0.0000,0.1467]
	33	0.2073	[0.1169,0.2977]
**The third round**			
No admixture VS Admixture	**11**	**0.5594**	**[0.4551,0.6637]**
	24	0.4406	[0.3363,0.5449]

Table S4 Posterior distributions of population demographic parameters from Scenario 11 with the highest posterior probability inferred in DIYABC analysis

Parameter	Mean	Median	Mode	Quantile 2.50%	Quantile 5.00%	Quantile 95.00%	Quantile 97.5%
N1	3.52×10^{3}	2.96×10^{3}	2.36×10^{3}	8.88×10^{2}	1.05×10^{3}	7.96×10^{3}	8.88×10^{3}
N2	1.16×10^{3}	9.56×10^{2}	8.34×10^{2}	3.57×10^{2}	4.25×10^{2}	2.46×10^{3}	3.57×10^{3}
N3	6.87×10^{2}	5.69×10^{2}	4.45×10^{2}	2.12×10^{2}	2.49×10^{2}	1.35×10^{3}	1.81×10^{3}
N4	1.58×10^{3}	1.32×10^{3}	9.47×10^{2}	4.47×10^{2}	5.34×10^{2}	3.67×10^{3}	5.02×10^{3}
t1	4.68×10^{3}	4.60×10^{3}	4.25×10^{3}	1.45×10^{3}	1.89×10^{3}	7.78×10^{3}	8.23×10^{3}
t2	5.90×10^{3}	5.90×10^{3}	5.80×10^{3}	2.57×10^{3}	3.06×10^{3}	8.74×10^{3}	9.13×10^{3}
t3	9.04×10^{3}	9.29×10^{3}	9.90×10^{3}	6.80×10^{3}	7.34×10^{3}	9.95×10^{3}	9.97×10^{3}
μmic	3.41×10^{-4}	2.99×10^{-4}	2.07×10^{-4}	1.40×10^{-4}	1.51×10^{-4}	6.66×10^{-4}	7.32×10^{-4}
pmic	2.75×10^{-1}	2.88×10^{-1}	3.00×10^{-1}	1.78×10^{-1}	2.05×10^{-1}	3.00×10^{-1}	3.00×10^{-1}
snimic	1.70×10^{-6}	4.86×10^{-7}	1.07×10^{-8}	1.27×10^{-8}	1.56×10^{-8}	7.42×10^{-6}	8.68×10^{-6}

N1, N2, N3 and N4, effective population sizes of the corresponding populations;

t1, divergence of JT20 from JT18 and GF20; t2, divergence of GF20 from DS19; t3, divergence of DS19 from JT18;

μmic, the mean mutation rate of SSR;

pmic, the mean distribution of the number of repeats of microsatellites;

snimic, the mean rate of single nucleotide insertions/deletions.
